# Exploring natural biodiversity to expand access to microbial terpene synthesis

**DOI:** 10.1186/s12934-019-1074-4

**Published:** 2019-02-01

**Authors:** Juan Rico, Katia Duquesne, Jean-Louis Petit, Aline Mariage, Ekaterina Darii, Frédéric Peruch, Véronique de Berardinis, Gilles Iacazio

**Affiliations:** 1 Aix-Marseille Univ, CNRS, Centrale Marseille, iSm2, Marseille, France; 2 CNRS, LCPO, UMR 5629, Univ. Bordeaux, Bordeaux INP, 33600 Pessac, France; 3 Génomique métabolique, Genoscope, Institut François Jacob, CEA, CNRS, Univ Evry, Univ Paris-Saclay, 91057 Evry, France

**Keywords:** IPK, Terpene biosynthesis, Neurosporene, Microbial production

## Abstract

**Background:**

Terpenes are industrially relevant natural compounds the biosynthesis of which relies on two well-established—mevalonic acid (MVA) and methyl erythritol phosphate (MEP)-pathways. Both pathways are widely distributed in all domains of life, the former is predominantly found in eukaryotes and archaea and the latter in eubacteria and chloroplasts. These two pathways supply isopentenyl diphosphate (IPP) and dimethylallyl diphosphate (DMAPP), the universal building blocks of terpenes.

**Results:**

The potential to establish a semisynthetic third pathway to access these precursors has been investigated in the present work. We have tested the ability of a collection of 93 isopentenyl phosphate kinases (IPK) from the biodiversity to catalyse the double phosphorylation of isopentenol and dimethylallyl alcohol to give, respectively IPP and DMAPP. Five IPKs selected from a preliminary in vitro screening were evaluated in vivo in an engineered chassis *E. coli* strain producing carotenoids. The recombinant pathway leading to the synthesis of neurosporene and lycopene, allows a simple colorimetric assay to test the potential of IPKs for the synthesis of IPP and DMAPP starting from the corresponding alcohols. The best candidate identified was the IPK from *Methanococcoides burtonii* (UniProt ID: Q12TH9) which improved carotenoid and neurosporene yields ~ 18-fold and > 45-fold, respectively. In our lab scale conditions, titres of neurosporene reached up to 702.1 ± 44.7 µg/g DCW and 966.2 ± 61.6 µg/L. A scale up to 4 L in-batch cultures reached to 604.8 ± 68.3 µg/g DCW and 430.5 ± 48.6 µg/L without any optimisation shown its potential for future applications. Neurosporene was almost the only carotenoid produced under these conditions, reaching ~ 90% of total carotenoids both at lab and batch scales thus offering an easy access to this sophisticated molecule.

**Conclusion:**

IPK biodiversity was screened in order to identify IPKs that optimize the final carotenoid content of engineered *E. coli* cells expressing the lycopene biosynthesis pathway. By simply changing the IPK and without any other metabolic engineering we improved the neurosporene content by more than 45 fold offering a new biosynthetic access to this molecule of upmost importance.

**Electronic supplementary material:**

The online version of this article (10.1186/s12934-019-1074-4) contains supplementary material, which is available to authorized users.

## Background

Terpenes are known as a large family of natural products with a broad range of complexities and functionalities [1, 2]. They possess many industrial applications in the fields of cosmetics, nutrition and pharmaceutics [3–5]. Neurosporene is a tetraterpene molecule that belongs to the widely commercialized carotenoid family of pigmented antioxidants. Other, commonly produced, members of this family are lycopene and beta-carotene. Unlike these, microbial production of neurosporene has been rarely described, probably because neurosporene is often a bound intermediate of lycopene synthase CrtI [6]. Like all terpenes, carotenoids are composed of C5 isoprene units and are naturally synthesized from the two common building blocks, isopentenyl diphosphate and dimethylallyl diphosphate (IPP and DMAPP). These two compounds are naturally synthesized through one of two possible and distinct routes, the mevalonic acid (MVA) pathway and the methyl erythritol phosphate (MEP) pathway. Both of these pathways have been extensively engineered for the production of a number of terpenes, including carotenoids, with different levels of success [7–9].

In this work, we propose an alternative way to produce IPP and DMAPP, the building blocks for terpenes. The capability of several putative isopentenyl phosphate kinases (IPKs) to produce these molecules from the industrially available and low cost precursors isopentenol and dimethylallyl alcohol (IOH and DMAOH) was measured. Many advantages can be expected from this production method for IPP and DMAPP. The biosynthesis of terpenes can be decoupled from the central metabolism and thus will have less impact on the production of biomass, since glucose (or another carbon source) can be reserved for the primary metabolism. Moreover, the need to produce (or overproduce) only one enzyme, the IPK, should lead to a simplification of any engineering study on this new route to the universal terpene precursors, DMAPP and IPP. In contrast, the natural pathways to synthesize these two compounds, the MEV and the MEP pathways, use 18 enzymes to transform glucose into DMAPP and IPP. An additional advantage of this route to terpenes is the possibility of controlling the ratio between DMAOH and IOH so as to adapt the method to the biosynthesis of either monoterpenes (C10, 1 DMAOH for 1 IOH), sesquiterpenes or triterpenes (C15 and C30, 1 DMAOH for 2 IOH), or diterpenes or tetraterpenes (C20 and C40, 1 DMAOH for 3 IOH). Finally, it provides an opportunity to regulate the addition of the carbon source (DMAOH, IOH) over time thus limiting the formation of DMAPP and IPP, compounds toxic to the cell [10].

We selected, using sequence-driven in silico approach, 93 IPKs from the available biodiversity, and the number of candidate IPKs was reduced to 5 after considering protein expression in *E. coli*, protein solubility and activity. The in vivo performance of these five most promising candidates was tested in *E. coli* cells bearing the full recombinant lycopene biosynthetic pathway. Around 18-fold improvement of total carotenoid biosynthesis was observed. It is noteworthy that while the full lycopene biosynthesis pathway was present, neurosporene was the most abundant terpenoid produced by the *E. coli* strains expressing IPKs, in culture medium supplemented with IOH and DMAOH. Carotenoid production reached a more than 45-fold specific improvement, with a ~ 90% relative abundance for neurosporene in the carotenoid fraction of cell extracts.

## Materials and methods

### Bacterial strains and chemicals

*Escherichia coli* DH5α was used for cloning and plasmid propagation. *Escherichia coli* BL21 (DE3) was used for protein expression and carotenoid biosynthesis. LB broth medium was purchased from Fisher Bioreagents™. Tryptone and yeast extract were from BD™.

Adenosine triphosphate (ATP), phosphoenolpyruvic acid monopotassium salt (PEP), reduced β-Nicotinamide adenine dinucleotide (NADH) and oxidized β-Nicotinamide adenine dinucleotide (NAD), isopropyl-β-d-thiogalactoside (IPTG), 2-(*N*-morpholino)ethanesulfonic acid (MES), the enzymes lactate dehydrogenase (LDH) and pyruvate kinase (PK) from rabbit muscle, glycerol, KH_2_PO_4_, K_2_HPO_4_, isopentenol, dimethylallyl alcohol, streptomycin, kanamycin, ampicillin and chloramphenicol were purchased from Sigma Aldrich (Millipore Sigma, St Louis, USA). Bovine serum albumin was purchased from Bio-Rad. Oligonucleotides were from Sigma-Aldrich (Millipore Sigma, St Louis, USA). *E. coli* strains BL21-CodonPlus (DE3)-RIPL were from Agilent technologies (Santa Clara, USA). Phusion^®^ High-Fidelity DNA Polymerase was from Fisher, T4 DNA ligase and restriction enzymes were provided by New England Biolabs^®^ Inc. and used according to manufacturer instructions.

### IPK collection from biodiversity

A sequence driven approach [11] was used with IPK experimentally described as references: IPK from *Thermoplasma acidophilum* (UniProt ID: Q9HLX1) [12], *Methanocaldococcus jannaschii* (UniProt ID: Q60352) [13], *Methanothermobacter thermautotrophicus* (UniProt ID: O26153) [12], *Haloferax volcanii* (UniProt ID: D4GWT7) [14]. Primers were chosen for 93 genes corresponding to the proteins selected from the UniProt database. Genes were cloned with a N-terminal histidine tag in a pET22b(+) (Novagen) modified for ligation independent cloning as already described [15]. All primers and strains are listed in Additional file 1: Table S1. All the bacterial strains were purchased from DSMZ collection. Each protein expression plasmid was transformed into *E. coli* BL21-CodonPlus (DE3)-RIPL. Cell culture, protein production and cell lysis was performed as previously published [15] in 96-well microplates. The lysis buffer contained Tris 50 mM pH 6.0, NaCl 50 mM, glycerol 10%, 0.2 µL Lysonase™ Bioprocessing Reagent (Novagen) and Pefabloc 1 mM. After centrifugation and recovery of the supernatant, the induction of the protein was checked using E-PAGE™ 8% Protein Gels, 48-well system from Invitrogen. Protein concentration was determined by the Bradford method, with bovine serum albumin as the standard (Bio-Rad). Gel analysis showed that, from 93 over-expressed proteins, 66 were correctly induced after IPTG addition but only 24 are always visible in cell free extract.

### Enzymatic screening by LC–MS analysis

Biochemical assays were performed in 96-well microplates. Enzyme assays were performed in a final volume of 100 µL containing MES buffer 50 mM pH 6.0, 10 mM of substrates (isopentenyl phosphate and dimethylallyl phosphate), ATP 2.5 mM, MgCl_2_ 5 mM, PEP 5 mM, pyruvate kinase 1U and cell lysate (0.05 to 0.1 mg/mL of total proteins). The coupled enzymatic system with pyruvate kinase was used in order to avoid potential ADP inhibition on kinase activity. Reactions were performed overnight at room temperature and then stopped by adding 1% TFA. After centrifugation, a sample was diluted 20 fold before LC/MS injection by transferring 10 µL of each well of acidified reaction media into 190 µL of mobile phase (80% acetonitrile and 20% aqueous phase 10 mM (NH_4_)_2_CO_3_) in a 96-well daughter microplate. Standards were prepared as described above replacing enzyme cell lysate by untransformed *E. coli* BL21 (DE3) cell lysate.

The detection of isopentenyl phosphate (IP) or dimethylallyl phosphate (DMAP) and isopentenyl diphosphate (IPP) or dimethylallyl diphosphate (DMAPP) was performed by LC/ESI–MS method using a Dionex UltiMate TCC-3000RS chromatographic system (Thermo Fisher Scientific, Courtaboeuf, France) coupled to a hybrid triple quadrupole-linear ion trap mass spectrometer (QTRAP 5500 from ABSciex, Courtaboeuf, France) equipped with a HESI source.

HPLC separation was achieved on a Sequant ZICpHILIC column 5 µm, 2.1 × 100 mm (Merck, Darmstadt, Germany) thermostated at 15 °C. Mobile phase flow rate was set at 0.2 mL/min, and injection volume was 3 μL. Phase A was an aqueous solution of 10 mM (NH_4_)_2_CO_3_ with the pH adjusted to 9.5 with NH_4_OH and organic phase B was acetonitrile. The following gradient conditions were applied: 0.5 min equilibration step at 80% of phase B; 4.5 min linear gradient from 80 to 40% of phase B; 2.5 min isocratic elution at 40% of phase B, return to 80% of phase B in 2 min and a reconditioning step of 6 min.

For mass spectrometry analysis, multiple reaction monitoring (MRM) in the negative ionization mode was applied. Three optimized MRM transitions were selected for IP (or DMAP): 245 → 79/227/63 and for IPP (or DMAPP): 165 → 79/97/63. The data processing was performed using Analyst software 1.5.1 (ABSciex). On each microtiter plate, *E. coli* cell lysate was used as the negative control. Enzymes were considered as positive when the signal was at least threefold higher than that of the negative control.

### In vivo expression of selected IPKs

The genes coding for the IPKs of *Thermoplasma acidophilum* (IPK-ta), *Methanococcus vannielii* (IPK-mv), *Methanolobus tindarius* (IPK-mt), *Methanosalsum zhilinae* (IPK-mz), *Methanococcus maripaludis* (IPK-mm) and *Methanococcoides burtonii* (IPK-mb) were amplified by PCR with their corresponding primers (Additional file 1: Table S2). The PCR products were subsequently restricted *Hin*dIII/*Not*I, except of that corresponding to IPK-mz which was restricted *Not*I/*Not*I, and subcloned into medium copy number vector pCDFDuet™-1 (20–40 copies per cell) and/or high copy number vector pRSFDuet™-1 (> 100 copies per cell) from Novagen linearized with the same enzyme(s) for in vivo experiments. Streptomycin and kanamycin were, respectively used to select transformants. All plasmids used for in vivo carotenoid production screening are listed in Additional file 1: Table S3.

### Culture media and growth conditions

Routine growth of *E. coli* strains was made in LB medium (1% casein peptone, 0.5% yeast extract, 1% NaCl) supplemented with 100 µg/mL ampicillin, 50 µg/mL kanamycin, 50 µg/mL streptomycin and/or 30 µg/mL chloramphenicol when appropriate. Cultures were performed at 37 °C and 1.5% agar was added to solid medium.

Carotenoid biosynthesis was conducted in 250 mL glass conical flasks containing 50 mL TB medium (1.2% tryptone, 2.4% yeast extract, 0.4% glycerol, 0.017 M KH_2_PO_4_, 0.072 M K_2_HPO_4_) supplemented with antibiotics at the same concentrations as for LB. Cultures were inoculated with cells from a 24 h preculture (5 mL LB; 37 °C; culture roller drum) to an initial cell density of ~ 0.05 at 600 nm and flasks were incubated at 30 °C; 200 rpm in a Multitron standard incubation shaker (Infors HT) for 24 h. Heterologous gene expression was induced at this point by addition of 1 mM IPTG. When required cultures were also supplemented at the same time with 18 mM isopentenol and 6 mM dimethylallyl alcohol. Control cultures without IPTG or substrates were grown in parallel for all transformants. After induction, cultures were incubated at 30 °C and 200 rpm for another 30 h. Cell pellets were then collected by centrifugation, washed and stored at − 20 °C until extraction. Dry cell weight was determined after desiccation at 70 °C until no further weight reduction was observed (24–48 h).

Batch fermentation was performed in a 7 L total volume (5.4 L working volume) BioBundle autoclavable glass bioreactor equipped with Rushton impellers—Applikon^®^ Biotechnology. Culture conditions were set and maintained with modules Bio controller ADI 1030 and Bio console ADI 1035—*Applikon*^®^ *Biotechnology*. Strains were inoculated at an OD_600_ ~ 0.05 in 4 L of TB with antibiotics and grown for 24 h (30 °C; 0.5 L/min aeration; 100 rpm agitation). After this period, the culture was supplemented (1 mM IPTG; 18 mM IOH; 6 mM DMAOH) and the agitation set to 150 rpm. The incubation was conducted for additional 76 h (100 h total fermentation time) and the cells collected by centrifugation.

### Sequence analysis and phylogenetic tree reconstruction

**Fig. 1:**
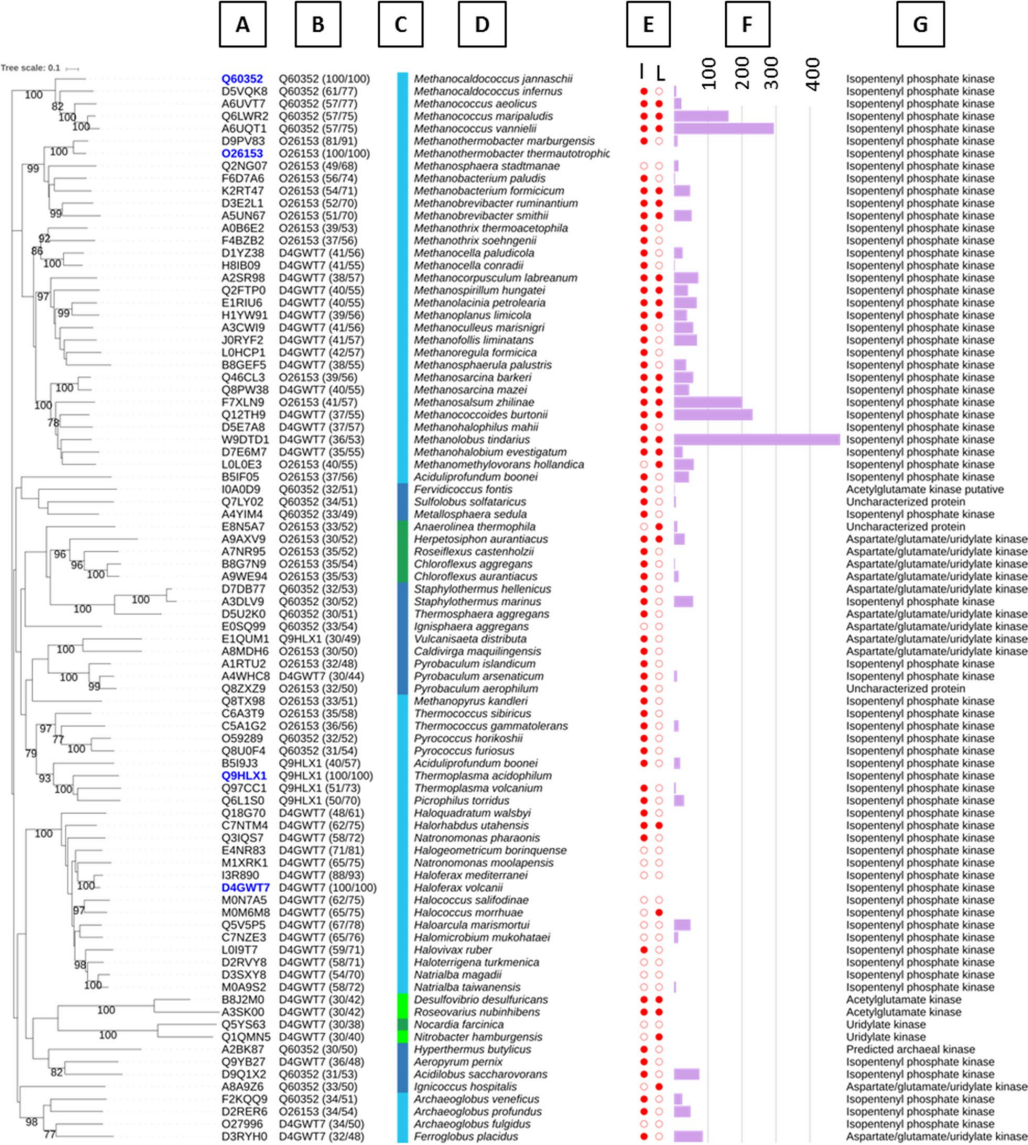
Phylogenetic tree of 82 cloned IPK candidates from biodiversity compared with IPKs from literature. Bootstrap values expressed as a percentage of 1000 replications are shown next to each node if value > 75%. **a** UniProt identifiers of each candidates. The 4 IPKs from literature used as the reference set in the sequence driven approach are noted in blue. **b** Best hit from IPK reference set and its percentage of identity and homology with the candidate. **c** Phylogenetic lineage of IPKs: Archaea are in blue (light blue: *Euryarchaeota*; dark blue: *Crenarchaeota*) and bacteria are in green (light green: *Proteobacteria*; dark green: *Terrabacteria* group). **d** Selected microorganisms. **e** Protein expression status according SDS gel analysis of cell free extract by eye. “I” full red dot means that the protein was visibly induced by IPTG treatment and “L” means that the protein is visibly present in the cell free extract. **f** Quantification by LC/MS analysis of IPP/DMAPP production (µM/L) in 96-microwell screening. **g** Protein name according to UniProt annotation

Pairwise sequence alignments were calculated with gapped BLASTp and the BLOSUM62 scoring matrix. Phylogenetic analysis (Fig. 1) was performed using MAFFT [16] for multiple sequence alignment, QUICKTREE [17] for tree building and iTOL [18] for tree rendering. Percent identity matrix was obtained after alignment with Clustal Omega online website (https://www.ebi.ac.uk/Tools/msa/clustalo/).

### Carotenoid extraction, analysis and quantification

Equal amounts of cells were resuspended in 2 mL deionized MQ water and extracted with 6 mL of 1:1:1 ethanol:acetone:petroleum ether. The mixture was vortexed to homogeneity and incubated for 30 min on ice. After the incubation period, the mixture was vortexed briefly and aqueous and organic phases were separated by centrifugation (6000 rpm; 10 min; 4 °C). Plate reader (Sinergy HTX–Biotek) was used to determine the absorbance spectrum (350–600 nm) of 100–200 µL of the petroleum ether phase in clear bottom 96-well microplates.

HPLC analysis was performed after evaporation of 1.5 mL of petroleum ether extract and re-solubilisation of carotenoids in 0.5 mL acetone. 10 µL of solubilized samples was analyzed by reverse phase HPLC on a Knauer Platin Blue system equipped with a diode array detector and a Macherey–Nagel Nucleodur C18-Gravity-SB column (100 mm × 2 mm, 1.8 mm), multi-detection at 415, 440, 470 and 505 nm was used. The eluent consisted of a 9:1 acetonitrile/THF mixture delivered at 0.35 mL/min. Retention times (lycopene 5.0 min and neurosporene 5.9 min) and calibration curves (at 470 nm) were calculated using pure lycopene and neurosporene standards provided by CaroteNature GmbH (Switzerland).

Batch fermentation samples were extracted several times with acetone (until no more carotenoids were remaining in the cell pellets). Acetone extracts were mixed together (~ 40 mL total volume) and extracted again with 10 mL petroleum ether. After phase separation (mediated by the addition of 10 mL distilled H_2_O and centrifugation) 0.5 mL of the petroleum ether phase was evaporated and carotenoids solubilised again in 0.5 mL acetone prior HPLC analysis.

## Results and discussion

### *Engineering E. coli chassis for* in vivo *optimisation of carotenoid production*

The implementation of a simple and reliable screening workflow to determine in vivo terpene production was the first milestone of this work. In order to facilitate this task, we decided to target carotenoid production due to their colour. Plasmids pACM-E_PAG_-B_PAG_ (carrying *crtE* and *crtB* genes from *Pantoea agglomerans*) and pUCM-I_RS_ (carrying *crtI* from *Rhodobacter sphaeroides*) have previously driven the accumulation of a mixture of lycopene and neurosporene of industrial interest when co-transformed into *E. coli* [19]. This short pathway took advantage of the natural synthesis of farnesyl diphosphate (FPP) from IPP and DMAPP by *E. coli.* We used these two plasmids to introduce this recombinant pathway into *E. coli* BL21 (DE3). After cultivation and solvent extraction of the modified strain absorption bands with maxima around 415, 440, 470 and 500 nm, matching those in the absorbance spectra of lycopene and neurosporene, were detected. No carotenoid production was observed in *E. coli* BL21 (DE3) strains co-transformed with empty plasmids pACYCDuet™-1 and pETDuet™-1 equivalent to those from which the full lycopene pathway is expressed (Additional file 1: Figure S1).

The use of enzymatic activities able to convert the C5 alcohols isopentenol (IOH) and/or dimethylallyl (DMAOH) into IPP and DMAPP may beoffer an alternative and/or a complementary way to enable microbial terpene synthesis avoiding competition with the biosynthesis of intermediates essential for *E. coli* fitness. The enzyme IPK from *Thermoplasma acidophilum* (herein IPK-ta) was able to catalyse the in vitro synthesis of DMAPP from DMAOH and to increase β-carotene biosynthesis by about 45% when this enzyme was overexpressed in *E. coli* cultures supplemented with 2 mM DMAOH [20].

To evaluate our chassis, the gene coding for IPK-ta was cloned into the commercial pCDFDuet™-1 backbone and the resulting plasmid (pCDF_IPK-ta) was co-transformed along with pACM-E_PAG_-B_PAG_ and pUCM-I_RS_ into *E. coli* BL21 (DE3). When supplemented with 1 mM IPTG, 6 mM DMAOH and 18 mM IOH, the cultured recombinant strain expressing both IPK-ta and the complete lycopene pathway led on average to an 85% increase in the absorbance at 470 nm relative to a non-supplemented culture of the same strain. We thus confirm the ability of IPK-ta to enhance the biosynthesis of carotenoids in the presence of alcohol substrates.

### Optimisation of IPK-ta expression to boost carotenoid production

It is generally accepted that increasing the number of copies of a gene may lead to higher expression levels and eventually protein accumulation and enzymatic activity. Therefore, we evaluated the performance of IPK-ta when expressed from the very high copy number plasmid pRSFDuet™-1. Plasmid pRSF_IPK–ta was co-transformed with pACM-E_PAG_-B_PAG_ and pUCM-I_RS_ into *E. coli* Bl21 (DE3). Spectrophotometric analysis of the solvent extracts showed a ninefold higher A_470_, from cultures with IPTG, IOH and DMAOH relative to the non-supplemented culture, much more than the less than twofold increase observed with the medium copy number plasmid pCDF_IPK-ta.

No increase in carotenoid production was observed after induction with IPTG in the absence of substrates or in control strains co-transformed with the empty backbone pRSFDuet™-1, pACM-E_PAG_-B_PAG_ and pUCM-I_RS_ (not shown). These results suggests that increasing the IPK-ta expression cassette copy number helps to increase IPK activity and thus the availability of terpene building blocks. Furthermore, the increase in carotenoid production was only about twofold when cultures were supplemented with 24 mM of only one substrate, either DMAOH or IOH, suggesting that the direct supply of the both terpene building blocks (IPP and DMAPP) improves carotenoid production (not shown).

### Exploring sequence diversity of IPK family

The results obtained with IPK-ta for carotenoid yield improvement starting from C5 alcohols DMAOH and IOH are promising. Nevertheless, the optimum temperature range of this enzyme is around 70 °C and it was just recently that this enzyme was shown to be able to convert DMAOH into DMAPP both in vitro and in vivo [20], being the first of its family reported to hold DMAOH phosphorylation activity. Therefore, it would be interesting to examine the natural biodiversity in the search of alternative candidates which optimum reaction conditions match better those generally found in industrial fermentation.

An IPK collection was then built by a sequence driven approach [11] using four experimentally described IPKs [12–14] as references (Fig. 1). 93 enzymes were selected from the UniProt database as representatives of the diversity. We cloned 82 genes and 64 were successfully over-expressed in *E. coli* BL21 (DE3). In order to identify active IPKs in our conditions, isopentenyl/dimethylallyl phosphate kinase activity at room temperature were assayed in cell free extracts using a mixture of isopentenyl phosphate (IP) and dimethylallyl phosphate (DMAP) as substrates and mass spectrometry for product analysis. We detected 39 active enzymes and selected five of them for further study based on IPK activities (Fig. 1f) and lack of similarity to IPK-ta (Fig. 1b). If, like IPK-ta, these enzymes show promiscuous kinase activities on IOH/DMAOH, the IP/DMAP formed by a first phosphorylation can be rapidly transformed by the same active enzymes into the corresponding diphosphates IPP and DMAPP.

These five selected IPKs (IPKs from *Methanococcus vanielii* (IPK-mv), *Methanolobus tindarius* (IPK-mt), *Methanosalsum zhilinae* (IPK-mz), *Methanococcus maripaludis* (IPK-mm) and *Methanococcoides burtonii* (IPK-mb)) were then included in the workflow to assess in vivo carotenoid production improvement starting from IOH and DMAOH.

### In vivo carotenoid production improvement by alternative IPKs

**Fig. 2:**
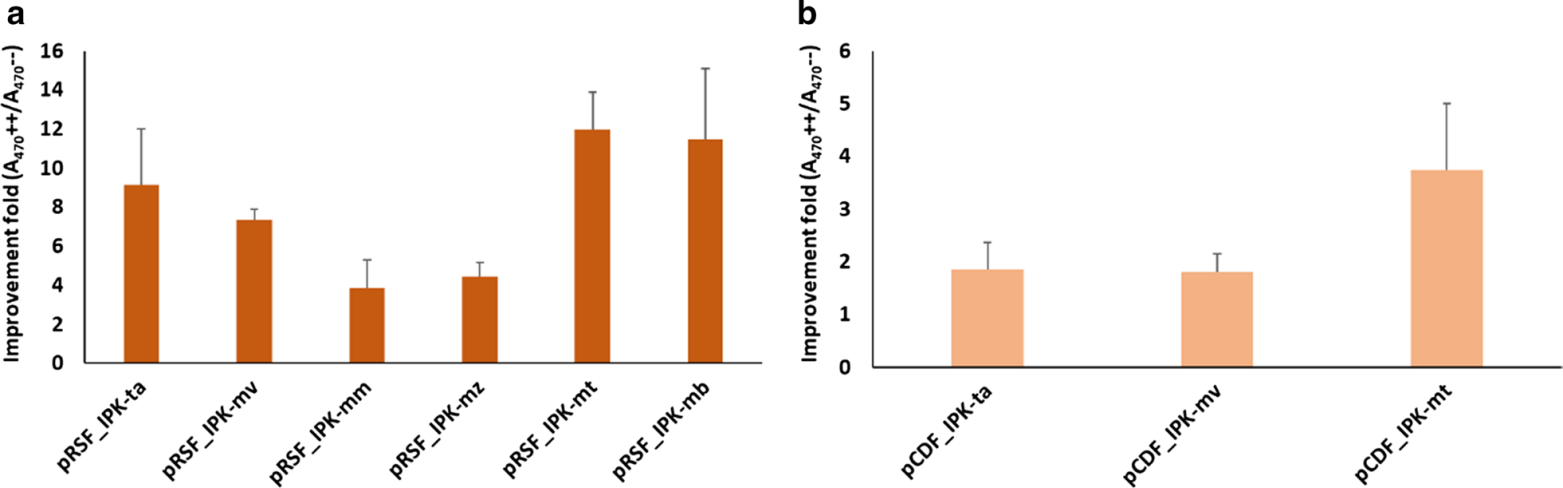
Screening of in vivo carotenoid production in *E. coli* expressing pACM-E_PAG_-B_PAG_ and pUCM-I_RS_ background. **a** Fold increases observed by expressing each of the six different IPKs from high copy pRSFDuet™-1. **b** Folds increases observed by expressing IPK-ta, IPK-mv and IPK-mt from medium copy pCDFDuet™-1. Results are expressed as the average and standard deviation of assays performed with at least two independent transformants

Carotenoid production of *E.coli* BL21 (DE3) strains carrying one of the plasmids pRSF_IPK-mt, pRSF_IPK-mv, pRSF_IPK-mb, pRSF_IPK-mm and pRSF_IPK-mz together with both pACM-E_PAG_-B_PAG_ and pUCM-I_RS_ was assessed. Analysis of the absorbance spectra revealed increased carotenoid production with each of the five new IPKs (IPK-mt: 12.0 ± 1.9; IPK-mv: 7.4 ± 0.5; IPK-mb: 11.5 ± 3.6; IPK-mm: 3.9 ± 1.4 and IPK-mz: 4.4 ± 0.7) (Fig. 2a). The maximum increases were obtained with IPK-mt and IPK-mb, respectively 12.0 and 11.5 fold.

**Fig. 3:**
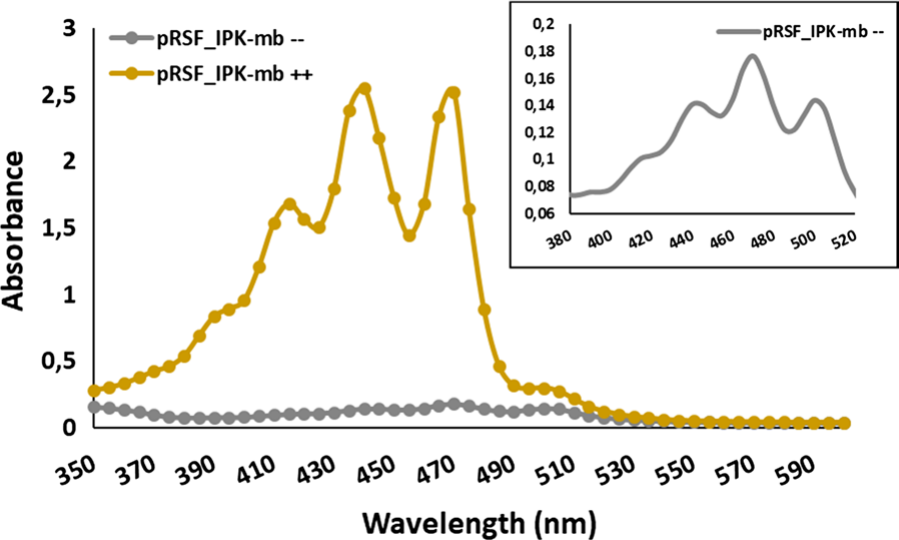
Comparison of the absorbance spectra obtained from *E. coli* BL21 (DE3) transformed with pRSF_IPK-mb, pACM-E_PAG_-B_PAG_ and pUCM-I_RS_ with (++) and without (−−) substrates supplementation. Zoom in of the spectrum without substrates shown in the right upper corner

Two phylogenetically distant IPKs, IPK-mv and IPK-mt (Fig. 1), that share only 33% identity, were also cloned in pCDFDuetTM-1 to confirm the backbone effect on the production of carotenoids. Plasmids pCDF_IPK-mt and pCDF_IPK-mv were co-transformed along with the full lycopene pathway in *E. coli* and carotenoid yields improvements obtained were 3.7 ± 1.3 and 1.81 ± 0.34, respectively (Fig. 2b). The highest copy number plasmid, pRSFDuetTM-1, remained the best performer for all tested IPKs and was chosen for subsequent experiments. No improvement was observed when protein expression was induced in the absence of substrates. In addition, as observed for IPK-ta, the improvement was limited in cultures supplemented with 24 mM DMAOH or 24 mM IOH in absence of the other substrate (not shown). This last observation supports the idea that the supplementation of the culture with the two alcohols substrates is more efficient to generate IPP and DMAPP than the supplementation of only one of the two. One of the causes of this improvement could be the circumvention of the endogenous isopentenyl diphosphate isomerase activity or other factors likely to influence the pool and the ratio of the diphosphate. At this point, best improvement (> 11-fold) was achieved with IPK-mt and IPK-mb, that share 63% of identity, cloned in the high copy number pRSF plasmid. Nevertheless, only IPK from *Methanococcoides burtonii* (UniProt ID: Q12TH9) was chosen for deeper analysis. In particular, a detailed analysis of the absorption spectra revealed that, whenever carotenoid production increased by induction of IPKs in the presence of C5 alcohols, the intensity of the shortest wavelength bands increased when compared to the same bands obtained from the control cultures (Fig. 3), thus suggesting a neurosporene enrichment of the mixture of carotenoids.

### Analysis of carotenoid production by IPK from *Methanococcoides burtonii*

**Fig. 4:**
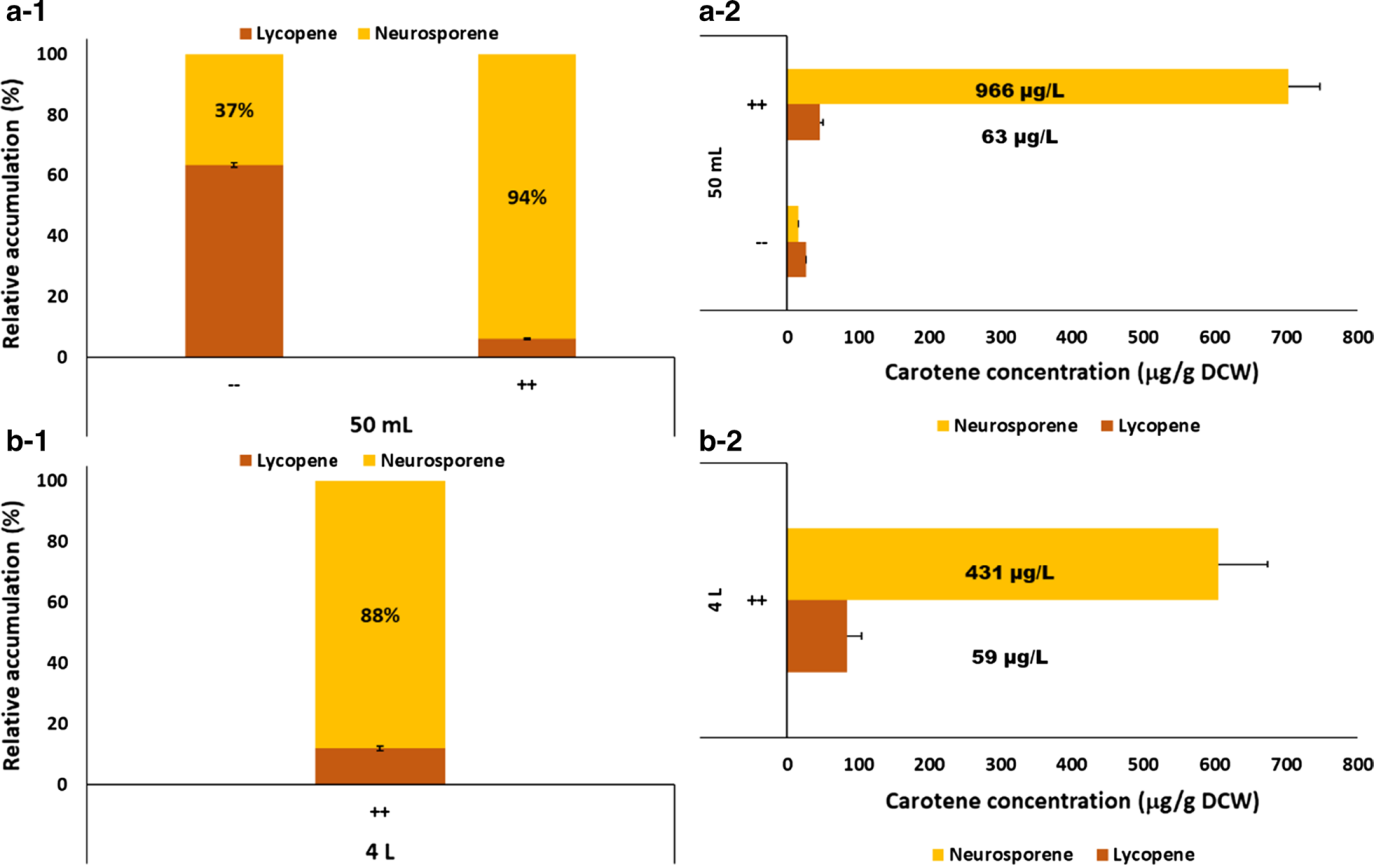
HPLC analysis of carotenoid lycopene and neurosporene production in *E. coli* BL21 (DE3) {pRSF_IPK-mb; pACM-E_PAG_-B_PAG_; pUCM-I_RS_}. **a**, **b** group 50 mL cultures and 4 L cultures experiments for carotenoid production, respectively. Sub-panel (1) for relative accumulation of lycopene and neurosporene. % of neurosporene accumulation is noted on the bars; (2) carotenoids concentration per cell weight with carotenoid concentration per liter written above the bars.). (−−) no substrates added (++) 18 mM IOH and 6 mM DMAOH added to the cultures

Carotenogenic transformants expressing IPK-mb were selected for further analysis of carotenoid production due to the good results obtained with our generic and simple in vivo screening method. The presence of lycopene (~ 63.5%) and neurosporene (~ 36.5%) in control cultures (without substrates) was confirmed by HPLC (Fig. 4a-1).

This result is similar to that described previously when using this set of carotenogenic enzymes [20]. Nonetheless, the extracts from cultures where IOH and DMAOH were supplemented (1 mM IPTG; 6 mM DMAOH and 18 mM IOH) showed a clear enrichment in neurosporene content (~ 93.9%) (Fig. 4a-1 and Additional file 1: Figure S2). Carotenoid concentration was calculated by interpolating the area of the lycopene and neurosporene peaks within their respective calibration curves (Additional file 1: Figure S3). Average concentration in supplemented cultures was 45.6 ± 4.5 µg/g DCW lycopene and 702.1 ± 44.7 µg/g DCW for neurosporene (Fig. 4a-2). This carotenoid yield comes because of improvement folds of 17.9 ± 1.7 for overall carotenoid content (lycopene + neurosporene) and 46.0 ± 5.2 when considering only neurosporene. These results confirmed the enrichment in neurosporene (up to almost 94% of the total carotenoids produced), revealing a specific neurosporene improvement of > 45-fold. The fact that CrtI from *R. sphaeroides* is a leaky phytoene dehydrogenase (naturally releasing both lycopene and neurosporene) was probably key reason for achieving this level of enrichment in neurosporene contents. It is yet to be discovered, to the best of our knowledge, an enzyme that produces specifically neurosporene as pure final product so this result could help to close the gap towards microbial synthesis of this high-value product minimizing downstream processing. It should be interesting to explore the sequence diversity of this family to find more efficient CrtI to improve this process.

### Scale-up in 4 L batch culture

All those results, even if yet far from direct application at industrial scale, might help opening a little more the door for alternative and/or complementary strategies for terpene backbones production and more concretely neurosporene. Next step undertaken was then to check the scalability of the process. *E. coli* BL21 (DE3) {pRSF_IPK-mb; pACM-E_PAG_-B_PAG_; pUCM-I_RS_} was inoculated at an OD_600_ ~ 0.05. After this period, batch culture was supplemented (1 mM IPTG; 18 mM IOH; 6 mM DMAOH) and conducted for additional 76 h (100 h total fermentation time) and the cells pelleted and extracted. HPLC analysis of these samples revealed that the carotenoid composition was ~ 12% lycopene and ~ 88% neurosporene (Fig. 4b-1) corresponding to the accumulation of 83.1 ± 20.6 µg/g DCW lycopene and 604.8 ± 68.3 µg/g DCW neurosporene (Fig. 4b-2). Both the concentration per cell and the purity of neurosporene are quite conserved under the newly tested conditions confirming the potential scalability of the process.

## Discussion

A number of interesting conclusions could be drawn from the results described herein. First, we show that archaebacterial IPKs, although evolutionarily distant, retain the ability to catalyze the synthesis of IPP and DMAPP using isopentenol and dimethylallyl alcohol as substrates, which facilitates in vivo access to carotenoids. In doing so, growth could be decoupled from terpene production reducing the potential burden to the main metabolism and biomass production. The fact that just one enzyme is enough to access both IPP and DMAPP from the corresponding prenols greatly simplifies the engineering task when compared to the use of MVA or MEP that use (in total) 18 enzymes to convert glucose to the diphosphates. The simplicity to express a single IPK makes it also not just an alternative if not an a priori complementary approach to further optimize terpene production of strains with any or both of these pathways pre-engineered. Furthermore, the ratio of IPP to DMAPP, that should be 3 to 1 for carotenoid synthesis, could be fixed easily at the start of the experiment by adding the adequate proportion of the two alcohols during fermentation and eventually adapted depending on the chain length of the target terpene. When compared to a previous report and according to our own results it is worth to use a mixture of prenols to conduct carotenoid biosynthesis. The best carotenoid yield observed in our work is more than 1 mg/L (mixture of neurosporene and lycopene) establishing the basis for future works dedicated to microbial neurosporene production. It should be stated that there is yet a huge space for improvement to fully exploit the potential of this strategy to access terpene building blocks as many microbiological, genetic and metabolic approaches among others could be applied to optimize the performance described in this work. Not only is this strategy compatible with the engineering of other pathways if not that its simplicity makes it easily transferrable to a number of hosts commonly used as workhorse in metabolic engineering (i.e. yeasts, fungi). It is finally of note that neurosporene is obtained as a major compound in our case. The inefficiency of the final enzyme to convert neurosporene into lycopene may provide us with a tool to biosynthesize the former nearly pure. This compound of medical interest as UV-B protectant [21] has so far no specific enzyme described for its production and is only biosynthesized as product of inefficient CrtI desaturase like the one from *R. sphaeroides* [22] employed in this study. In this work, we established IPKs as biocatalysts to convert both C5 prenols IOH and DMAOH into their diphosphate derivatives and thus established a semisynthetic green access to terpenes starting from cheap, easy-to-access substrates.

## Conclusions

By screening IPK diversity, we were able to improve carotenoid production in *E. coli* cells bearing only the final lycopene biosynthetic path (CrtE, CrtB and CrtI) as well as IPK as recombinant enzymes. Maximum improvements of ~ 18-fold and > 45-fold were obtained for total carotenoid and neurosporene yields, respectively. Titres of neurosporene reached up to 702 µg/gDCW corresponding to 966 µg/L at lab scale without any metabolic engineering providing access to such a valuable compound. Medium engineering, semi-continuous IOH and DMAOH feeding as well as increased biomass yield are expected to improve final yield in neurosporene.

## Additional file

**Additional file 1: Table S1.** IPK collection from biodiversity. **Table S2.** Oligonucleotides used in this study for IPK cloning in pCDFDuetTM-1 and/or pRSFDuet™-1. **Table S3.** List of plasmids used in this study for in vivo screening of IPKs. **Figure S1.** Absorbance spectrums of carotenoid extracts. Solvent (plain line), *E. coli* BL21 (DE3) transformed with pACYCDuet™-1 and pETDuet™-1 (round marker) and pACM-E_PAG_-B_PAG_ and pUCM-I_RS_ (pCarot - diamond marker). **Figure S2.** HPLC analysis of carotenoids. **A** Pure neurosporene. **B** Extract from *E. coli* cells transformed with *ipk*-*mb*. **C** Pure lycopene. **Figure S3.** Calibration curves and absorbance spectrums of pure carotenoids. Up-left: HPLC calibration curve of lycopene at 470 nm (X-axis: area of the peak generated at the corresponding retention time; Y-axis: ng of compound injected). Up-right: HPLC calibration curve of neurosporene at 470 nm. Bottom: Absorbance spectrums of pure solutions of lycopene and neurosporene.
