# Supplementary material for: Exploring natural biodiversity to expand access to microbial terpene synthesis
Source: Microb Cell Fact. 2019 Feb 1;18:23. doi: 10.1186/s12934-019-1074-4 (PMC6359773; doi:10.1186/s12934-019-1074-4)
Supplement: Supplementary file 1 — Additional file 1: Table S1. IPK collection from biodiversity. Table S2. Oligonucleotides used in this study for IPK cloning in pCDFDuetTM-1 and/or pRSFDuet™-1. Table S3. List of plasmids used in this study for in vivo screening of IPKs. Figure S1. Absorbance spectrums of carotenoid extracts. Solvent (plain line), E. coli BL21 (DE3) transformed with pACYCDuet™-1 and pETDuet™-1 (round marker) and pACM-EPAG-BPAG and pUCM-IRS (pCarot - diamond marker). Figure S2. HPLC analysis of carotenoids. A Pure neurosporene. B Extract from E. coli cells transformed with ipk-mb. C Pure lycopene. Figure S3. Calibration curves and absorbance spectrums of pure carotenoids. Up-left: HPLC calibration curve of lycopene at 470 nm (X-axis: area of the peak generated at the corresponding retention time; Y-axis: ng of compound injected). Up-right: HPLC calibration curve of neurosporene at 470 nm. Bottom: Absorbance spectrums of pure solutions of lycopene and neurosporene. [file 12934_2019_1074_MOESM1_ESM.docx]

**Table S1.** IPK collection from biodiversity.

| **Selected UniProt Entry** | **Organism of the selected protein** | **Strain used for gene PCR amplification** | **Primers** | |
| --- | --- | --- | --- | --- |
|  |  |  | **Fw primer** | **Rev primer** |
| A3DLV9 | *Staphylothermus marinus* (strain ATCC 43588 / DSM 3639 / JCM 9404 / F1) | DSMZ-3639 | AAAGAAGGAGATAGGATCATGCATCATCACCATCACCATAATACTAGCAGTGATCAT | GTGTAATGGATAGTGATCTTAGCTCATATCTATCACTGT |
| A5UN67 | *Methanobrevibacter smithii* (strain ATCC 35061 / DSM 861 / OCM 144 / PS) | DSMZ-861 | AAAGAAGGAGATAGGATCATGCATCATCACCATCACCATATTATTTTAAAAATTGGAGG | GTGTAATGGATAGTGATCTTATCCCCTTGAAATAACAGTTCCC |
| A6UQT1 | *Methanococcus vannielii* (strain ATCC 35089 / DSM 1224 / JCM 13029 / OCM 148 / SB) | DSMZ-1224 | AAAGAAGGAGATAGGATCATGCATCATCACCATCACCATTTTGCAATTTTAAAACTTGG | GTGTAATGGATAGTGATCTTAATTTATTGAAGTTCCTTTTACATT |
| A6UVT7 | *Methanococcus aeolicus* (strain ATCC BAA-1280 / DSM 17508 / OCM 812 / Nankai-3) | DSMZ-17 | AAAGAAGGAGATAGGATCATGCATCATCACCATCACCATTTGGCCATTTTAAAACTTGGAG | GTGTAATGGATAGTGATCTTAATTAATTTTAGTTCCATTAACCTC |
| B5I9J3 | *Aciduliprofundum boonei* (strain DSM 19572 / T469) | DSMZ-19572 | AAAGAAGGAGATAGGATCATGCATCATCACCATCACCATCTCCTTGTTAAACTCGG | GTGTAATGGATAGTGATCTTATTCAACCACCGTCCCTATG |
| B5IF05 | *Aciduliprofundum boonei* (strain DSM 19572 / T469) | DSMZ-19572 | AAAGAAGGAGATAGGATCATGCATCATCACCATCACCATGCCTGGAGTACATACG | GTGTAATGGATAGTGATCTTACTTTATTTTTATTTTGGTGAATTTTGC |
| D3E2L1 | *Methanobrevibacter ruminantium* (strain ATCC 35063 / DSM 1093 / JCM 13430 / OCM 146 / M1) | DSMZ-1093 | AAAGAAGGAGATAGGATCATGCATCATCACCATCACCATATTATTTTAAAAATTGGCGG | GTGTAATGGATAGTGATCTTATTCCTTGGAAATTTTAGTTCCTCTAAC |
| D5VQK8 | *Methanocaldococcus infernus* (strain DSM 11812 / JCM 15783 / ME) | DSMZ-11812 | AAAGAAGGAGATAGGATCATGCATCATCACCATCACCATTTAGCCATCTTAAAGTTAGGGG | GTGTAATGGATAGTGATCTTATTGGGGATCTATTAAGGTTCCTAC |
| D7DB77 | *Staphylothermus hellenicus* (strain DSM 12710 / JCM 10830 / BK20S6-10-b1 / P8) | DSMZ-12710 | AAAGAAGGAGATAGGATCATGCATCATCACCATCACCATAGTATTAATAGGGATCAC | GTGTAATGGATAGTGATCTTAGCTCATATCTATTACAGTACCCCTCAATAC |
| D7E6M7 | *Methanohalobium evestigatum* (strain ATCC BAA-1072 / DSM 3721 / NBRC 107634 / OCM 161 / Z-7303) | DSMZ-3721 | AAAGAAGGAGATAGGATCATGCATCATCACCATCACCATAGCACAAATTCATCCG | GTGTAATGGATAGTGATCTTATTGCCCTGAACCTGTAATCG |
| E0SQ99 | *Ignisphaera aggregans* (strain DSM 17230 / JCM 13409 / AQ1.S1) | DSMZ-17230 | AAAGAAGGAGATAGGATCATGCATCATCACCATCACCATGGAATAACAATTAATATAGTTAAAATTGGAG | GTGTAATGGATAGTGATCTTATAGCATTATTCTTGTTCCTTCAACAC |
| F6D7A6 | *Methanobacterium paludis* (strain DSM 25820 / JCM 18151 / SWAN1) | DSMZ-25820 | AAAGAAGGAGATAGGATCATGCATCATCACCATCACCATATAATCCTAAAACTCGGTGGAAG | GTGTAATGGATAGTGATCTTATTTTTTTTTACTTTTAATCAAAG |
| F7XLN9 | *Methanosalsum zhilinae* (strain DSM 4017 / NBRC 107636 / OCM 62 / WeN5) | DSMZ-4017 | AAAGAAGGAGATAGGATCATGCATCATCACCATCACCATAGTCAACATAAAAACATTACAATTCTG | GTGTAATGGATAGTGATCTTATTTTTTTATTGCAGTTCCTA |
| I0A0D9 | *Fervidicoccus fontis* (strain DSM 19380 / JCM 18336 / VKM B-2539 / Kam940) | DSMZ-19380 | AAAGAAGGAGATAGGATCATGCATCATCACCATCACCATAAAGTCGTTATAAAGCTTGGAGG | GTGTAATGGATAGTGATCTTATGGAATTATTTTTGTTCCATTGAC |
| Q2NG07 | *Methanosphaera stadtmanae* (strain ATCC 43021 / DSM 3091 / JCM 11832 / MCB-3) | DSMZ-3091 | AAAGAAGGAGATAGGATCATGCATCATCACCATCACCATATAATACTAAAAATAGGTGGAAGTGCAATAAC | GTGTAATGGATAGTGATCTTATTTTATAATTGTACCTTTAACTTTTTGTCC |
| Q6L1S0 | *Picrophilus torridus* (strain ATCC 700027 / DSM 9790 / JCM 10055 / NBRC 100828) | DSMZ-9790 | AAAGAAGGAGATAGGATCATGCATCATCACCATCACCATATCATAGTAAAGCTTGGTGGC | GTGTAATGGATAGTGATCTTATTCTATCACCGAACCTATAAAATCATC |
| Q6LWR2 | *Methanococcus maripaludis* (strain S2 / LL) | DSMZ-14266 | AAAGAAGGAGATAGGATCATGCATCATCACCATCACCATTTTGCAATCTTAAAACTAGGCG | GTGTAATGGATAGTGATCTTAGTTTATCAATGTTCCTTTTACATT |
| Q7LY02 | *Sulfolobus solfataricus* (strain ATCC 35092 / DSM 1617 / JCM 11322 / P2) | DSMZ-1617 | AAAGAAGGAGATAGGATCATGCATCATCACCATCACCATGAAATGGATATGGGATCTG | GTGTAATGGATAGTGATCTTAGGCATTCGGATTACCTCTTAC |
| Q97CC1 | *Thermoplasma volcanium* (strain ATCC 51530 / DSM 4299 / JCM 9571 / NBRC 15438 / GSS1) | DSMZ-4299 | AAAGAAGGAGATAGGATCATGCATCATCACCATCACCATATAATAATCAAACTAGGTGGAAGCC | GTGTAATGGATAGTGATCTTACTTTATCACCGTTCCCGTG |
| A0A090I163 | *Methanobacterium formicicum* | DSMZ-3637 | AAAGAAGGAGATAGGATCATGCATCATCACCATCACCATATTATCCTTAAACTCGGCGGG | GTGTAATGGATAGTGATCTTATTTCCGTATAATAGTTCCCCGA |
| A0B6E2 | *Methanothrix thermoacetophila* (strain DSM 6194 / JCM 14653 / NBRC 101360 / PT) | DSMZ-6194 | AAAGAAGGAGATAGGATCATGCATCATCACCATCACCATCTTAAGATACTGAAGCTTGGCGG | GTGTAATGGATAGTGATCTTATCTACCTGTGATGAGGGTGCCG |
| A1RTU2 | *Pyrobaculum islandicum* (strain DSM 4184 / JCM 9189 / GEO3) | DSMZ-4184 | AAAGAAGGAGATAGGATCATGCATCATCACCATCACCATTATATAGTCAAATTTGGCGGTTCTGC | GTGTAATGGATAGTGATCTTAAGGCTCAGCCATGGTGCAACTC |
| A2BK87 | *Hyperthermus butylicus* (strain DSM 5456 / JCM 9403 / PLM1-5) | DSMZ-5456 | AAAGAAGGAGATAGGATCATGCATCATCACCATCACCATCCTCGAGGCGGGGCT | GTGTAATGGATAGTGATCTTAGGGGAGTATTTCAGTGCCAACC |
| A2SR98 | *Methanocorpusculum labreanum* (strain ATCC 43576 / DSM 4855 / Z) | DSMZ-4855 | AAAGAAGGAGATAGGATCATGCATCATCACCATCACCATAGTGAACTGGTAATTATCAAACTCGGC | GTGTAATGGATAGTGATCTTATTTTTTCACCAGAGTTCCG |
| A4YIM4 | *Metallosphaera sedula* (strain ATCC 51363 / DSM 5348 / JCM 9185 / NBRC 15509 / TH2) | DSMZ-5348 | AAAGAAGGAGATAGGATCATGCATCATCACCATCACCATGGCAAGCCTAATCGAGTTATTAAAC | GTGTAATGGATAGTGATCTTATCTACTCACCTTGATCAAC |
| A8A9Z6 | *Ignicoccus hospitalis* (strain KIN4/I / DSM 18386 / JCM 14125) | DSMZ-18386 | AAAGAAGGAGATAGGATCATGCATCATCACCATCACCATATAGTGATAAAGTTAGGGGGCAGCG | GTGTAATGGATAGTGATCTTATCCTTGTCGACAAGGTAC |
| A8MDH6 | *Caldivirga maquilingensis* (strain ATCC 700844 / DSM 13496 / JCM 10307 / IC-167) | DSMZ-13496 | AAAGAAGGAGATAGGATCATGCATCATCACCATCACCATATATTAATTAAGCTGGGTGGATCAGC | GTGTAATGGATAGTGATCTTACGTAGCCTCAATTCTAGTGCATCCC |
| A9AXV9 | *Herpetosiphon aurantiacus* (strain ATCC 23779 / DSM 785) | DSMZ-785 | AAAGAAGGAGATAGGATCATGCATCATCACCATCACCATAATAAGCCTATTTTTATCAAAC | GTGTAATGGATAGTGATCTTAATCAAGCTTGATGATCGTG |
| A9WE94 | *Chloroflexus aurantiacus* (strain ATCC 29366 / DSM 635 / J-10-fl) | DSMZ-635 | AAAGAAGGAGATAGGATCATGCATCATCACCATCACCATTATACCTTCATCAAATTTGG | GTGTAATGGATAGTGATCTTACCATCTCTTTATCACCGTGCCC |
| B8G7N9 | *Chloroflexus aggregans* (strain MD-66 / DSM 9485) | DSMZ-9485 | AAAGAAGGAGATAGGATCATGCATCATCACCATCACCATTATACCTTCGTCAAATTCGGCGGT | GTGTAATGGATAGTGATCTTATTGCTTGATGAGCGTACCGTTTG |
| B8GEF5 | *Methanosphaerula palustris* (strain ATCC BAA-1556 / DSM 19958 / E1-9c) | DSMZ-19958 | AAAGAAGGAGATAGGATCATGCATCATCACCATCACCATCATAATCAGGTGATGCTGAAACTC | GTGTAATGGATAGTGATCTTACGCCATCCTGACCACAGTCC |
| C5A1G2 | *Thermococcus gammatolerans* (strain DSM 15229 / JCM 11827 / EJ3) | DSMZ-15229 | AAAGAAGGAGATAGGATCATGCATCATCACCATCACCATATAATAGTCAAGATCGGGGGTAGCG | GTGTAATGGATAGTGATCTTAATGTACAATCCTCGTCCCAAATCC |
| C6A3T9 | *Thermococcus sibiricus* (strain MM 739 / DSM 12597) | DSMZ-12597 | AAAGAAGGAGATAGGATCATGCATCATCACCATCACCATATAATAATCAAACTCGGCGGAAGTG | GTGTAATGGATAGTGATCTTACCACCTCTTAATAACCGTCCCAGG |
| D1YZ38 | *Methanocella paludicola* (strain DSM 17711 / JCM 13418 / NBRC 101707 / SANAE) | DSMZ-17711 | AAAGAAGGAGATAGGATCATGCATCATCACCATCACCATACGACCATACTCAAGATCGGC | GTGTAATGGATAGTGATCTTAGTCTCCGCGTATTATTGTGCCTTC |
| D2RER6 | *Archaeoglobus profundus* (strain DSM 5631 / JCM 9629 / NBRC 100127 / Av18) | DSMZ-5631 | AAAGAAGGAGATAGGATCATGCATCATCACCATCACCATATAGTAGTCAAGATTGGTGGATCTG | GTGTAATGGATAGTGATCTTAGTCAGATTTAACGAGCGTTCC |
| D3RYH0 | *Ferroglobus placidus* (strain DSM 10642 / AEDII12DO) | DSMZ-10642 | AAAGAAGGAGATAGGATCATGCATCATCACCATCACCATAAGATTTTGAAGATTGGAGGTTCTTTG | GTGTAATGGATAGTGATCTTAGAGCTTGTAATTTCCTTTTTCAATTT |
| D5E7A8 | *Methanohalophilus mahii* (strain ATCC 35705 / DSM 5219 / SLP) | DSMZ-5219 | AAAGAAGGAGATAGGATCATGCATCATCACCATCACCATAATGAGAAAAACAGCGATATTACC | GTGTAATGGATAGTGATCTTAAGCCTTGTCTTTAATTGCAGTACC |
| D5U2K0 | *Thermosphaera aggregans* (strain DSM 11486 / M11TL) | DSMZ-11486 | AAAGAAGGAGATAGGATCATGCATCATCACCATCACCATACCAGGGACGTGGTGTTTC | GTGTAATGGATAGTGATCTTAAACTATTTCAATAATCGTGGCCTTC |
| D9PV83 | *Methanothermobacter marburgensis* (strain ATCC BAA-927 / DSM 2133 / JCM 14651 / NBRC 100331 / OCM 82 / Marburg) | DSMZ-2133 | AAAGAAGGAGATAGGATCATGCATCATCACCATCACCATATCATTCTCAAGGTAGGTGGAAGTG | GTGTAATGGATAGTGATCTTATTTTCTGATCCTGGTGCCCCTC |
| E1QUM1 | *Vulcanisaeta distributa* (strain DSM 14429 / JCM 11212 / NBRC 100878 / IC-017) | DSMZ-14429 | AAAGAAGGAGATAGGATCATGCATCATCACCATCACCATAGGCTTTTCATGATAAAGTT | GTGTAATGGATAGTGATCTTAATCAGATAAAAACCTCGTGC |
| E1RIU6 | *Methanolacinia petrolearia* (strain DSM 11571 / OCM 486 / SEBR 4847) | DSMZ-11571 | AAAGAAGGAGATAGGATCATGCATCATCACCATCACCATAAAGAGAAGATTATATTAAAGCTGGG | GTGTAATGGATAGTGATCTTAAGGCGACACCCTTGTGC |
| E8N5A7 | *Anaerolinea thermophila* (strain DSM 14523 / JCM 11388 / NBRC 100420 / UNI-1) | DSMZ-14523 | AAAGAAGGAGATAGGATCATGCATCATCACCATCACCATAGCATGGACTCCAATCTGACTTTTC | GTGTAATGGATAGTGATCTTATGAGTTTCGAGAGAGGATTTTCGTTC |
| F2KQQ9 | *Archaeoglobus veneficus* (strain DSM 11195 / SNP6) | DSMZ-11195 | AAAGAAGGAGATAGGATCATGCATCATCACCATCACCATAAGATTGATGGCCTGAGAA | GTGTAATGGATAGTGATCTTACAGCCTGATTTTGGTGCCTACG |
| F4BZB2 | *Methanothrix soehngenii* (strain ATCC 5969 / DSM 3671 / JCM 10134 / NBRC 103675 / OCM 69 / GP-6) | DSMZ-3671 | AAAGAAGGAGATAGGATCATGCATCATCACCATCACCATGTTCGGGTCTTGAAGATC | GTGTAATGGATAGTGATCTTAATCCGGCCTCCAGACCTTG |
| H1YW91 | *Methanoplanus limicola* DSM 2279 | DSMZ-2279 | AAAGAAGGAGATAGGATCATGCATCATCACCATCACCATACAGTTAAAGAAATTATTATTTT | GTGTAATGGATAGTGATCTTAATGGTCTTTGGTCACCAC |
| H8IB09 | *Methanocella conradii* (strain DSM 24694 / JCM 17849 / CGMCC 1.5162 / HZ254) | DSMZ-24694 | AAAGAAGGAGATAGGATCATGCATCATCACCATCACCATACGATAATACTCAAGATTGGCGG | GTGTAATGGATAGTGATCTTAGTCTCCGCTTATGAGTGTGCCTTC |
| K2RT47 | *Methanobacterium formicicum* (strain DSM 3637 / PP1) | DSMZ-3637 | AAAGAAGGAGATAGGATCATGCATCATCACCATCACCATATTATCCTGAAACTGGGTGGAAGTG | GTGTAATGGATAGTGATCTTATTGTTTACTTATAATTGTTCCCCTGAC |
| L0L0E3 | *Methanomethylovorans hollandica* (strain DSM 15978 / NBRC 107637 / DMS1) | DSMZ-15978 | AAAGAAGGAGATAGGATCATGCATCATCACCATCACCATGTTTCATCGGAAAGTCTAACC | GTGTAATGGATAGTGATCTTAAGACCTGCATTCATATTGTTCATGTG |
| O27996 | *Archaeoglobus fulgidus* (strain ATCC 49558 / VC-16 / DSM 4304 / JCM 9628 / NBRC 100126) | DSMZ-4304 | AAAGAAGGAGATAGGATCATGCATCATCACCATCACCATTCAGAATTGAAGAGTGACGTTACCA | GTGTAATGGATAGTGATCTTAGATTATCACCTCCGTTCCAACCTTC |
| O59289 | *Pyrococcus horikoshii* (strain ATCC 700860 / DSM 12428 / JCM 9974 / NBRC 100139 / OT-3) | DSMZ-12428 | AAAGAAGGAGATAGGATCATGCATCATCACCATCACCATATAATAATAAAGATTGGAGGTAGCG | GTGTAATGGATAGTGATCTTAAGGAACGACAACGGTGC |
| Q12TH9 | *Methanococcoides burtonii* (strain DSM 6242 / NBRC 107633 / OCM 468 / ACE-M) | DSMZ-6242 | AAAGAAGGAGATAGGATCATGCATCATCACCATCACCATAACTCAAATAACGGGATAAC | GTGTAATGGATAGTGATCTTATGAATCTTTTATTGCGGTACCGATG |
| Q18G70 | *Haloquadratum walsbyi* (strain DSM 16790 / HBSQ001) | DSMZ-16790 | AAAGAAGGAGATAGGATCATGCATCATCACCATCACCATTCAAGAGAAACTAACTCAC | GTGTAATGGATAGTGATCTTACTGAATGCTTGTTCCAACTTGTTTATG |
| Q2FTP0 | *Methanospirillum hungatei* JF-1 (strain ATCC 27890 / DSM 864 / NBRC 100397 / JF-1) | DSMZ-864 | AAAGAAGGAGATAGGATCATGCATCATCACCATCACCATCCTGATCGAATCATTTTAAAACTTGGC | GTGTAATGGATAGTGATCTTATGCTCCCTCCGGCAGG |
| Q46CL3 | *Methanosarcina barkeri* (strain Fusaro / DSM 804) | DSMZ-804 | AAAGAAGGAGATAGGATCATGCATCATCACCATCACCATAATGTCTCAACAGAACCTGTTATTC | GTGTAATGGATAGTGATCTTATACCCTTTTGTCCGGACTGATTGTG |
| Q8PW38 | *Methanosarcina mazei* (strain ATCC BAA-159 / DSM 3647 / Goe1 / Go1 / JCM 11833 / OCM 88) | DSMZ-3647 | AAAGAAGGAGATAGGATCATGCATCATCACCATCACCATAATGCTTCAAACGAACCTGTTATCC | GTGTAATGGATAGTGATCTTATACCCTTTTATCCGGACTGATCCTG |
| Q8U0F4 | *Pyrococcus furiosus* (strain ATCC 43587 / DSM 3638 / JCM 8422 / Vc1) | DSMZ-3638 | AAAGAAGGAGATAGGATCATGCATCATCACCATCACCATATCCTTGTAAAAATCGGAGGAAGTG | GTGTAATGGATAGTGATCTTAAGGTCTAACAATAGTCCCGATACC |
| Q8ZXZ9 | *Pyrobaculum aerophilum* (strain ATCC 51768 / IM2 / DSM 7523 / JCM 9630 / NBRC 100827) | DSMZ-7523 | AAAGAAGGAGATAGGATCATGCATCATCACCATCACCATTACGTTATCAAATTCGGCG | GTGTAATGGATAGTGATCTTAAAGGGCGGGCAGCCC |
| Q9V186 | *Pyrococcus abyssi* (strain GE5 / Orsay) | DSMZ-25543 | AAAGAAGGAGATAGGATCATGCATCATCACCATCACCATATAATAGTTAAAATAGGGGG | GTGTAATGGATAGTGATCTTAAGATTGGACAACCGTTCCAATTCC |
| U1NEE4 | *Haloquadratum walsbyi* J07HQW2 | DSMZ-16790 | AAAGAAGGAGATAGGATCATGCATCATCACCATCACCATTCAGAGGAAACTGAGGAAATATCTG | GTGTAATGGATAGTGATCTTAATTAACTGTTGTTCCGACATCTTCGC |
| W9DTD1 | *Methanolobus tindarius* DSM 2278 | DSMZ-2278 | AAAGAAGGAGATAGGATCATGCATCATCACCATCACCATGATAACAATAATATTACTATCC | GTGTAATGGATAGTGATCTTATATAGTCCCGGCTCCTAT |
| A3CWI9 | *Methanoculleus marisnigri* (strain ATCC 35101 / DSM 1498 / JR1) | DSMZ-1498 | AAAGAAGGAGATAGGATCATGCATCATCACCATCACCATACTGAGAGAGTGGTACTGAAAC | GTGTAATGGATAGTGATCTTATGGTTTACTCCTTGCGGTTATCG |
| A3SK00 | *Roseovarius nubinhibens* (strain ATCC BAA-591 / DSM 15170 / ISM) | DSMZ-15170 | AAAGAAGGAGATAGGATCATGCATCATCACCATCACCATAACCGTGACTGGATTGCCACCGC | GTGTAATGGATAGTGATCTTACTTTGCACGGATAATTGAACCGGCCCC |
| A4WHC8 | *Pyrobaculum arsenaticum* (strain DSM 13514 / JCM 11321) | DSMZ-13514 | AAAGAAGGAGATAGGATCATGCATCATCACCATCACCATCACATAATCAAATTCGGAGGTAG | GTGTAATGGATAGTGATCTTATCTTGTTAGTGTGGGCTCCACGACGG |
| A7NR95 | *Roseiflexus castenholzii* (strain DSM 13941 / HLO8) | DSMZ-13941 | AAAGAAGGAGATAGGATCATGCATCATCACCATCACCATATCACATTCATCAAGTGGGGCGGTTCC | GTGTAATGGATAGTGATCTTAACCTGCAACCATCACCGTCCCCTCG |
| B1YAP2 | *Pyrobaculum neutrophilum* (strain DSM 2338 / JCM 9278 / V24Sta) | DSMZ-2338 | AAAGAAGGAGATAGGATCATGCATCATCACCATCACCATTATATCGTAAAATTCGGAGGCTC | GTGTAATGGATAGTGATCTTAGGGCTCCACGGCGGTACAG |
| B8J2M0 | *Desulfovibrio desulfuricans* (strain ATCC 27774 / DSM 6949) | DSMZ-6949 | AAAGAAGGAGATAGGATCATGCATCATCACCATCACCATAACAACGCCACGGTTGTCATCAAA | GTGTAATGGATAGTGATCTTAGCCGATGACCACGGTTCCTAGGGGG |
| D3SXY8 | *Natrialba magadii* (strain ATCC 43099 / DSM 3394 / CIP 104546 / JCM 8861/ NBRC 102185 / NCIMB 2190 / MS3) | ATCC-43099 | AAAGAAGGAGATAGGATCATGCATCATCACCATCACCATATCGTTCTGAAACTCGGCGGCAGCG | GTGTAATGGATAGTGATCTTAGCGAACCGTCGTCCCGGGCTC |
| D8J317 | *Halalkalicoccus jeotgali* (strain DSM 18796 / CECT 7217 / JCM 14584 / KCTC 4019 / B3) | DSMZ-18796 | AAAGAAGGAGATAGGATCATGCATCATCACCATCACCATATCGTCCTGAAACTCGGCGGGTCG | GTGTAATGGATAGTGATCTTAGTCGATCCGGGTCCCGGCG |
| D9Q1X2 | *Acidilobus saccharovorans* (strain DSM 16705 / JCM 18335 / VKM B-2471 / 345-15) | DSMZ-16705 | AAAGAAGGAGATAGGATCATGCATCATCACCATCACCATAGGTGCAACCTAGCTGTAGTC | GTGTAATGGATAGTGATCTTAGGGCACGACCCTAGTGCCTACCTCC |
| E4NR83 | *Halogeometricum borinquense* (strain ATCC 700274 / DSM 11551 / JCM 10706 / PR3) | DSMZ-11551 | AAAGAAGGAGATAGGATCATGCATCATCACCATCACCATACGACCGTCCTCAAACTCGG | GTGTAATGGATAGTGATCTTAATCACGGATGACGGTTCCGGCG |
| I3R890 | *Haloferax mediterranei* (strain ATCC 33500 / DSM 1411 / JCM 8866 / NBRC 14739 / NCIMB 2177 / R-4) | DSMZ-1411 | AAAGAAGGAGATAGGATCATGCATCATCACCATCACCATAGCCTCGTCGTCCTCAAACTCGGC | GTGTAATGGATAGTGATCTTATTCCCCGCGAATCACGGTGCC |
| J0RYF2 | *Methanofollis liminatans* DSM 4140 | DSMZ-4140 | AAAGAAGGAGATAGGATCATGCATCATCACCATCACCATAATAGAGAGATTACACTGCTGAAG | GTGTAATGGATAGTGATCTTATCTTCTGACGATCGTTCCGCTGTGG |
| L0HCP1 | *Methanoregula formicica* (strain DSM 22288 / NBRC 105244 / SMSP) | DSMZ-22288 | AAAGAAGGAGATAGGATCATGCATCATCACCATCACCATTCTGAAAGGGTACTTTTAAAACTC | GTGTAATGGATAGTGATCTTACTCTCCTCTTACGCTCGTCCCGCC |
| L0I9T7 | *Halovivax ruber* (strain DSM 18193 / JCM 13892 / XH-70) | DSMZ-18193 | AAAGAAGGAGATAGGATCATGCATCATCACCATCACCATACGGTCGTCCTGAAACTCGGCGG | GTGTAATGGATAGTGATCTTAGTCGATCGTCGTTCCCGGATCGTCCC |
| L9X3Q1 | *Natronococcus amylolyticus* DSM 10524 | DSMZ-10524 | AAAGAAGGAGATAGGATCATGCATCATCACCATCACCATATCGTCCTGAAACTCGGCGGCAG | GTGTAATGGATAGTGATCTTATTCGATCGTCGTCCCCGCCCC |
| M0A9S2 | *Natrialba taiwanensis* DSM 12281 | DSMZ-12281 | AAAGAAGGAGATAGGATCATGCATCATCACCATCACCATATCGTCCTGAAACTCGGCGGCAG | GTGTAATGGATAGTGATCTTACTGCACCGTCGTTCCCGGGTTCTC |
| M0M6M8 | *Halococcus morrhuae* DSM 1307 | DSMZ-1307 | AAAGAAGGAGATAGGATCATGCATCATCACCATCACCATGAGCGAAGCGGAATGACCGT | GTGTAATGGATAGTGATCTTAGCCGACGCGCGTCCCGGG |
| M0MRH4 | *Halococcus saccharolyticus* DSM 5350 | DSMZ-5350 | AAAGAAGGAGATAGGATCATGCATCATCACCATCACCATACCACCGTCCTCAAGCTCGGGGGAA | GTGTAATGGATAGTGATCTTATCCGACCCGGGTTCCCGGCGATTC |
| M0N7A5 | *Halococcus salifodinae* DSM 8989 | DSMZ-8989 | AAAGAAGGAGATAGGATCATGCATCATCACCATCACCATACGACCGTCCTCAAACTCGG | GTGTAATGGATAGTGATCTTATCCGTCCGTTCGGATCTTCGTTCCTGG |
| M1XRK1 | *Natronomonas moolapensis* (strain DSM 18674 / JCM 14361 / 8.8.11) | DSMZ-18674 | AAAGAAGGAGATAGGATCATGCATCATCACCATCACCATACGCCGGAGCAAGACGCGC | GTGTAATGGATAGTGATCTTACTCTATTTCGGTCCCGGGGTC |
| Q1QMN5 | *Nitrobacter hamburgensis* (strain DSM 10229 / NCIMB 13809 / X14) | DSMZ-10229 | AAAGAAGGAGATAGGATCATGCATCATCACCATCACCATGATGAGCCGGCCTATCGTCGTGTTG | GTGTAATGGATAGTGATCTTAACTGGCCACGATCGTTCCTCG |
| Q3IQS7 | *Natronomonas pharaonis* (strain ATCC 35678 / DSM 2160 / CIP 103997 /OS NBRC 14720 / NCIMB 2260 / Gabara) | DSMZ-2160 | AAAGAAGGAGATAGGATCATGCATCATCACCATCACCATACGACAGTACTGAAGCTCGGCGG | GTGTAATGGATAGTGATCTTACCCGACTCGCGTTCCGGGGTTC |
| Q5V5P5 | *Haloarcula marismortui* (strain ATCC 43049 / DSM 3752 / JCM 8966 / VKM B-1809) | DSMZ-3752 | AAAGAAGGAGATAGGATCATGCATCATCACCATCACCATACGGTCGTCCTCAAACTCGGCGG | GTGTAATGGATAGTGATCTTAATCGGCGTCATCACCAGCGATAGTCGT |
| Q5YS63 | *Nocardia farcinica* (strain IFM 10152) | DSMZ-43665 | AAAGAAGGAGATAGGATCATGCATCATCACCATCACCATACGGACCCGGGTACCGACC | GTGTAATGGATAGTGATCTTAGGAACGAACCAATGTGCCGATCTTCTC |
| Q8TX98 | *Methanopyrus kandleri* (strain AV19 / DSM 6324 / JCM 9639 / NBRC 100938) | DSMZ-6324 | AAAGAAGGAGATAGGATCATGCATCATCACCATCACCATTTCACGGTTCTGAAGCTCGGAGGG | GTGTAATGGATAGTGATCTTAGCGCGTGATCCTCGTACCAACGTG |
| Q9YB27 | *Aeropyrum pernix* (strain ATCC 700893 / DSM 11879 / JCM 9820 / NBRC 100138 / K1) | DSMZ-11879 | AAAGAAGGAGATAGGATCATGCATCATCACCATCACCATGCTAGCCCAGGATCCCGGG | GTGTAATGGATAGTGATCTTAACGCGCATCAACCCTGGTCC |
| B0R4Z2 | *Halobacterium salinarum* (strain ATCC 29341 / DSM 671 / R1) | DSMZ-671 | AAAGAAGGAGATAGGATCATGCATCATCACCATCACCATACCGTCGTTGTCAAGCTCGGCG | GTGTAATGGATAGTGATCTTAGCCGTTGATCCGCGTTCCCAC |
| B9LPX1 | *Halorubrum lacusprofundi* (strain ATCC 49239 / DSM 5036 / JCM 8891 / ACAM 34) | DSMZ-5036 | AAAGAAGGAGATAGGATCATGCATCATCACCATCACCATAGCGCGGATCGCGGGGCG | GTGTAATGGATAGTGATCTTAGTCGATCCGCGTTCCGGCAT |
| C7NTM4 | *Halorhabdus utahensis* (strain DSM 12940 / JCM 11049 / AX-2) | DSMZ-12940 | AAAGAAGGAGATAGGATCATGCATCATCACCATCACCATACGACGATCCTCAAACTCGGGGGC | GTGTAATGGATAGTGATCTTACTCGATGAGCGTGCCCGGATTCTCG |
| C7NZE3 | *Halomicrobium mukohataei* (strain ATCC 700874 / DSM 12286 / JCM 9738 / NCIMB 13541) | DSMZ-12286 | AAAGAAGGAGATAGGATCATGCATCATCACCATCACCATAGTCCCGAGACGGTCGTCCTGAAA | GTGTAATGGATAGTGATCTTACTCCGGACCACCGGCGATC |
| D2RVY8 | *Haloterrigena turkmenica* (strain ATCC 51198 / DSM 5511 / NCIMB 13204 / VKM B-1734) | DSMZ-5511 | AAAGAAGGAGATAGGATCATGCATCATCACCATCACCATATCGTCCTGAAACTTGGCGGCAGCG | GTGTAATGGATAGTGATCTTACGTCTCCGACGAGTCGATCG |
| E4NA59 | *Kitasatospora setae* (strain ATCC 33774 / DSM 43861 / JCM 3304 / KCC A-0304 / NBRC 14216 / KM-6054) | DSMZ-43861 | AAAGAAGGAGATAGGATCATGCATCATCACCATCACCATAGCGCCCCGGCGGCGCC | GTGTAATGGATAGTGATCTTACCGGGTCTCCTTCCGGGGG |
| L9YZX9 | *Natrinema pallidum* DSM 3751 | DSMZ-3751 | AAAGAAGGAGATAGGATCATGCATCATCACCATCACCATATCGTGCTGAAACTCGGCGGCAG | GTGTAATGGATAGTGATCTTAGTCGATCGTCGTCCCCGGGTTCTCG |

**Table S2.** Oligonucleotides used in this study for IPK cloning in pCDFDuetTM-1 and/or pRSFDuet^TM^-1.

| **Primer name** | **Primer sequence 5’-3’** |
| --- | --- |
| *Hind*III_IPK-Ta_fw | CGACAAGCTTATGATGATACTGAAGATAG |
| *Not*I_IPK-Ta_rv | TATGCGGCCGCTCATCTTATCACCGTACCTATG |
| *Hind*III_IPK-Mv_fw | CGACAAGCTTATGTTTGCAATTTTAAAACTTGGCGGAAG |
| *Not*I_IPK-Mv_rv | TATGCGGCCGCTTAATTTATTGAAGTTCCTTTTACATTTTTTAAAAG |
| *Hind*III_IPK-Mt_fw | CGACAAGCTTATGGATAACAATAATATTACTATCCTGAAGATAG |
| *Not*I_IPK-Mt_rv | TATGCGGCCGCTTATATAGTCCCGGCTCCTATTG |
| *Not*I_IPK-Mz_fw | TATGCGGCCGCATGAGTCAACATAAAAACATTACAATTC |
| *Not*I_IPK-Mz_rv | TATGCGGCCGCTTATTTTTTTATTGCAGTTCCTATG |
| *Hind*III_IPK-Mm_fw | CGACAAGCTTATGTTTGCAATCTTAAAACTAGGCGG |
| *Not*I_IPK-Mm_rv | TATGCGGCCGCTTAGTTTATCAATGTTCCTTTTAC |
| *Hind*III_IPK-Mb_fw | CGACAAGCTTATGAACTCAAATAACGGGATAAC |
| *Not*I_IPK-Mb_rv | TATGCGGCCGCTTATGAATCTTTTATTGCGGTACC |

**Table S3**. List of plasmids used in this study for *in vivo* screening of IPKs.

| **Plasmid name** | **Relevant properties (ori); [copy number]; {antibiotic resistance}** | **Source or reference** |
| --- | --- | --- |
| pCDFDuet^TM^-1 | (CloDF13); [20–40]; {streptomycin} | Novagen |
| pRSFDuet^TM^-1 | (RSF1030); [> 100]; {kanamycin} | Novagen |
| pACYCDuet^TM^-1 | (P15A); [10–12]; {chloramphenicol} | Novagen |
| pETDuet^TM^-1 | (ColE1); [~40]; {ampicillin} | Novagen |
| pACM-E_PAG_-B_PAG_ | Constitutive expression of *crtE* and *crtB* genes from *P. agglomerans*; (P15A); [10–12]; {chloramphenicol} | Song et al., 2013 |
| pUCM-I_RS_ | Constitutive expression of *crtI* gene from *R.sphaeroides*; (pMB1); [> 100]; {ampicillin} | Song et al., 2013 |
| pCDF_IPK-ta | Inducible expression of IPK from *T. acidophilum*; (CloDF13); [20–40]; {streptomycin} | This study |
| pRSF_IPK-ta | Inducible expression of IPK from *T. acidophilum*; (RSF1030); [> 100]; {kanamycin} | This study |
| pCDF_IPK-mv | Inducible expression of IPK from *M. vanielii*; (CloDF13); [20–40]; {streptomycin} | This study |
| pRSF_IPK-mv | Inducible expression of IPK from *M. vanielii*; (RSF1030); [> 100]; {kanamycin} | This study |
| pCDF_IPK-mt | Inducible expression of IPK from *M. tindarius*; (CloDF13); [20–40]; {streptomycin} | This study |
| pRSF_IPK-mt | Inducible expression of IPK from *M. tindarius*; (RSF1030); [> 100]; {kanamycin} | This study |
| pRSF_IPK-mb | Inducible expression of IPK from *M. burtonii*; (RSF1030); [> 100]; {kanamycin} | This study |
| pRSF_IPK-mz | Inducible expression of IPK from *M. zhilinae*; (RSF1030); [> 100]; {kanamycin} | This study |
| pRSF_IPK-mm | Inducible expression of IPK from *M. maripaludis*; (RSF1030); [> 100]; {kanamycin} | This study |

**Figure S1.** **Absorbance spectrums of carotenoid extracts.** Solvent (plain line), *E. coli* BL21 (DE3) transformed with pACYCDuet^TM^-1 and pETDuet^TM^-1 (round marker) and pACM-E_PAG_-B_PAG_ and pUCM-I_RS_ (pCarot - diamond marker).


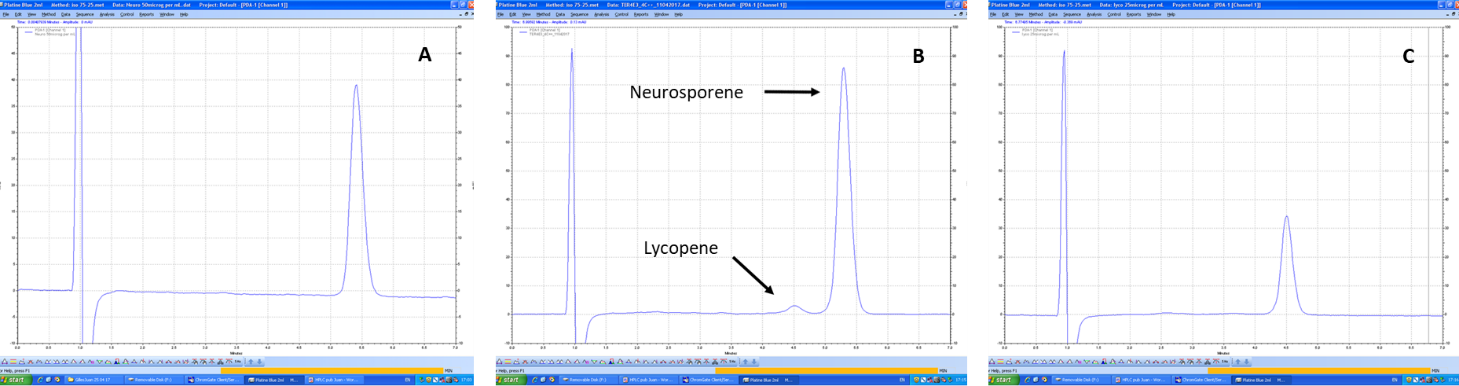


**Figure S2. HPLC analysis of carotenoids. A** Pure neurosporene. **B** Extract from *E. coli* cells transformed with *ipk-mb*. **C** Pure lycopene.

**Figure S3. Calibration curves and absorbance spectrums of pure carotenoids.** Up-left: HPLC calibration curve of lycopene at 470 nm (X-axis: area of the peak generated at the corresponding retention time; Y-axis: ng of compound injected). Up-right: HPLC calibration curve of neurosporene at 470 nm. Bottom: Absorbance spectrums of pure solutions of lycopene and neurosporene.
